# WSI-Babel-Shark: A modular pipeline for robust metadata extraction from heterogeneous whole slide image labels in digital pathology

**DOI:** 10.1016/j.jpi.2026.100722

**Published:** 2026-09-24

**Authors:** Shahram Aliyari, Maximilian Legnar, Christoph Blattgerste, Ayk Jessen, Balamurugan Thirukonda Subramanian Balakrishnan, Nils Englert, Philip S.J. Kaib, Mark Sementsov, Constantin Schwab, Cleo-Aron Weis

**Affiliations:** a Section Computational Pathology Heidelberg, Institute of Pathology Heidelberg, University Hospital Heidelberg, University of Heidelberg, Heidelberg, Germany; b Interdisciplinary Center for Scientific Computing (IWR), Heidelberg University, Heidelberg, Germany; c Data Analysis and Modeling in Medicine, Mannheim Institute for Intelligent Systems in Medicine (MIISM), Heidelberg University, Mannheim, Germany

**Keywords:** Digital pathology, Whole-slide imaging, Metadata extraction, Label analysis, Optical character recognition, Automation

## Abstract

**Introduction:**

Automated extraction of slide metadata remains a major bottleneck in digital pathology, especially in settings where barcode identifiers are missing, damaged, or inconsistent. Manual transcription of case, block, and stain information is error-prone and does not scale. Building on our previous whole-slide image (WSI)-Babel-Fish framework, we developed WSI-Babel-Shark, a more extensible pipeline for reliable metadata extraction across heterogeneous slide-label layouts.

**Materials and methods:**

WSI-Babel-Shark integrates data matrix code decoding, region of interest (ROI)-based optical character recognition (OCR), and rule-based metadata parsing within a modular Python and Docker architecture. To handle diverse institutional label formats, the system incorporates a deep learning-based label layout classifier using a ResNet-18 backbone, fused with handcrafted descriptors and prototype-based open-set recognition to categorize labels into 10 predefined layout groups. A graphical interface supports ROI annotation, dataset management, and configuration editing. Performance was evaluated on 270 WSIs (.svs and .ndpi formats) and cross-validated using 110 slides from the previously published WSI-Babel-Fish dataset to assess backward compatibility.

**Results:**

The label layout classifier achieved an overall accuracy of 97.4%, with macro-averaged precision, recall, and F1-score of 0.98 while reliably rejecting previously unseen layouts. End-to-end pipeline evaluation achieved complete SlideID reconstruction for 258 of 267 evaluable WSIs (96.6%). Independent comparison of OCR-derived metadata with the corresponding Data Matrix-decoded metadata on 236 slides demonstrated complete metadata concordance for 230 slides (97.5%), whereas field-level concordance ranged from 98.3% to 100.0% across all evaluated metadata fields. Cross-dataset analysis confirmed full compatibility with WSI-Babel-Fish label structures.

**Conclusion:**

WSI-Babel-Shark advances automated metadata extraction from WSIs by introducing a label-layout classification framework validated on heterogeneous label layouts and incorporating open-set recognition, together with standardized ROI annotation and a fully containerized implementation. The framework ensures reproducible linkage between digitized slides and lab information, is designed to facilitate interoperability with lab information systems, and provides a scalable foundation for large-scale digital pathology workflows. The complete implementation is openly available at: https://github.com/cpheidelberg/WSI-Babel-Shark.

## Highlights

• A modular pipeline for robust metadata extraction from heterogeneous slide labels.
• Hybrid barcode–OCR workflow improves accuracy across multi-scanner environments.
• Deep layout classifier enables reliable ROI selection and open-set rejection.
• Standardized SlideID synthesis ensures reproducible linkage across digital workflows.
• Containerized design supports scalable deployment and institution-agnostic integration.

## Introduction and objectives

Reliable slide-level metadata are essential for modern digital pathology workflows. They enable the linkage of whole-slide images (WSIs) with corresponding cases in lab information systems (LIS) and picture archiving and communication systems (PACS). Accurate metadata ensure traceability, facilitate automated data integration, and support downstream analyses as well as FAIR data stewardship.^1^, ^2^ In routine diagnostic practice, barcode-based identifiers typically provide this linkage by encoding case and block information directly on slide labels, allowing scanners and LIS interfaces to synchronize records automatically.^3^ However, when barcode identifiers are missing, unreadable, or inconsistent across systems, automated linkage becomes non-trivial and requires robust text-based extraction from label images.

In research, archival, and biobank settings, barcode-based identifiers are often missing, damaged, or do not align with institutional standards. The problem is further compounded by the high variability of label designs across scanners and institutions. Differences in typography, label orientation, and the presence of handwritten elements pose major challenges for optical character recognition (OCR)-based metadata extraction.^4^ Several open-source frameworks provide essential infrastructure for digital pathology, such as OpenSlide for vendor-neutral WSI access^5^ and DICOM-based standards for metadata representation.^6^, ^7^ Yet, only a few recent approaches have addressed the practical challenge of automatically extracting structured metadata from heterogeneous label layouts. Whereas some OCR-based methods, such as AutoLDP,^8^ have demonstrated high accuracy for standardized label formats, most existing tools still rely on fixed spatial and semantic rules or manually defined regions of interest (ROIs), which limits their scalability and adaptability in multi-institutional settings.^4^, ^8^ Consequently, there remains a need for a metadata extraction framework that can automatically adapt to heterogeneous label layouts, detect previously unseen label structures, recover metadata when barcode information is incomplete or unavailable, and be designed for deployment across different scanner platforms and institutional workflows.

Our previous framework, WSI-Babel-Fish, demonstrated the feasibility of automated metadata extraction from label images using OCR.^4^ The system successfully retrieved case numbers, specimen identifiers (Pot identifier), block number, and staining information directly from label regions and achieved high accuracy within a single institutional context. However, despite these promising results, the WSI-Babel-Fish approach had several critical limitations. It relied on fixed label layouts, lacked the capability to classify or reject unseen label types, and could not automatically determine the label layout category to guide downstream ROI extraction. In addition, WSI-Babel-Fish required external software such as ImageJ^9^ for ROI definition and did not incorporate barcode-based redundancy. These constraints collectively limited its generalizability, maintainability, and integration potential across different scanners or institutions. Collectively, these limitations motivated the development of WSI-Babel-Shark as a flexible and extensible metadata extraction framework designed to accommodate heterogeneous label layouts across different scanners and institutions through hybrid label-layout classification, classifier-guided ROI localization, barcode-aware metadata extraction, and a modular software architecture.

To overcome these limitations, we developed WSI-Babel-Shark, a modular and extensible pipeline for robust metadata extraction from heterogeneous WSI labels. WSI-Babel-Shark integrates a deep learning-based label layout classifier capable of classifying known label formats while identifying previously unseen layouts through an open-set recognition strategy. It provides a dedicated ROI-annotation interface that generates JSON configurations, thereby eliminating the need for third-party tools. The system employs a hierarchical extraction strategy that prioritizes data matrix code decoding and, when necessary, applies ROI-based OCR with rule-based validation to ensure reliability. All modules are containerized for reproducible deployment and orchestrated through a unified configuration and logging framework. Extracted metadata and associated quality metrics are stored in an integrated SQLite database, ensuring full traceability and data integrity.

This study presents the design, implementation, and validation of WSI-Babel-Shark and compares its performance with the previously developed WSI-Babel-Fish framework. Unlike existing OCR-based metadata extraction approaches, WSI-Babel-Shark combines prototype-based open-set layout recognition, classifier-guided dynamic ROI localization, barcode-aware redundancy, rule-based metadata validation, and a modular containerized architecture within a unified framework. Together, these methodological advances provide a unified end-to-end framework for automated slide-label metadata extraction, addressing several practical limitations of previous OCR-based approaches while facilitating reproducible deployment. Rather than introducing a single algorithmic improvement, WSI-Babel-Shark integrates these complementary methodological advances into a unified end-to-end framework for automated slide-label metadata extraction, thereby addressing several practical limitations of existing OCR-based approaches within a single reproducible workflow.

## Materials and methods

This section outlines the datasets, architecture, and evaluation procedures used to develop and validate the WSI-Babel-Shark framework. We first describe the characteristics and sources of the datasets, followed by details of the modular pipeline architecture, including data matrix code decoding, layout classification, ROI-based OCR, and rule-based parsing. Subsequent subsections explain the classifier design, graphical annotation tools, and evaluation strategy used to assess performance, robustness, and reproducibility. The section concludes with implementation details and information on code and data availability.

### Slide data and source

All slides included in this study originated from routine diagnostic workflows conducted at the Institute of Pathology Heidelberg (IPH). No patient-identifying information is contained in these slides. For the publicly shared subset of data, all case identifiers were replaced with fully synthetic, non-patient identifiers to ensure that no identifiable information is disclosed. The dataset represents a small but diverse subset of the institute's digital pathology archive and includes a variety of tissue types, staining protocols, and label formats commonly encountered in daily diagnostic practice. The slides were digitized using two high-resolution scanners, the Hamamatsu NanoZoomer S60 and the Leica GT450, which produced WSIs in the standard digital formats (.ndpi and .svs). The IPH applies a consistent internal nomenclature for case and slide identification, which provides the structural basis for metadata extraction in the WSI-Babel-Shark framework.

Each WSI in .svs or .ndpi format contains both the scanned tissue section and the corresponding slide label area. The WSI-Babel-Shark pipeline automatically isolates this label region and converts it into a standardized image representation that preserves all printed and handwritten information on the physical slide. These label images form the primary input for the metadata extraction process, which focuses exclusively on the label content rather than the tissue section.

Compared with the dataset used in the earlier WSI-Babel-Fish study, the current dataset incorporates material from multiple scanner systems and includes labels with greater variability in layout, typography, and staining annotations. Details regarding the standard structure of slide labels and the associated naming conventions have been described previously in work on the WSI-Babel-Fish framework. In the present study, this variability was intentionally retained to evaluate the robustness and generalizability of WSI-Babel-Shark under realistic heterogeneous scanner and label-layout conditions.

Only the information visible on the slide labels, such as case numbers, block identifiers, and stain designations, was used in this study. The general structure of IPH slide labels and the corresponding naming conventions have been described in prior work and served as the reference for interpreting all metadata elements in the current dataset. No patient names, clinical information, or other personally identifiable data were accessed or processed. All analyses were performed in full compliance with the data protection and confidentiality regulations of the IPH and the General Data Protection Regulation (EU 2016/679).

### Datasets

Four distinct datasets were used for the development and evaluation of the WSI-Babel-Shark framework, each serving a specific methodological purpose. All datasets originated from the diagnostic archive of the IPH and were strictly partitioned to ensure complete separation between training, validation, and evaluation phases. No slide used for evaluation was included in the datasets employed for classifier training or in any material derived from the earlier WSI-Babel-Fish framework.

#### Primary dataset for classifier development

A total of 1836 slide-label images were collected and manually categorized into 10 distinct layout groups (G1–G10), representing the predominant label configurations routinely encountered within the institute. Because no universal ROI configuration could reliably capture metadata across the diverse label formats encountered in routine lab practice, these images were systematically reviewed. The review identified 10 recurring layout configurations, and labels were subsequently grouped according to the spatial arrangement of ROI-relevant elements, including the positions of the Data Matrix code, lab identifier, case identifier, block identifier, stain field, and other printed information. Although some layouts differed only in one or a few metadata regions, separate layout groups were intentionally maintained whenever these differences affected ROI definition. This conservative grouping strategy enabled the assignment of a dedicated ROI template to each layout group, thereby ensuring accurate localization of metadata fields before OCR. As a result, 10 recurring layout configurations were identified, collectively representing all routinely encountered label structures within the available dataset. Each layout group was subsequently associated with a dedicated JSON-based ROI template used for automated metadata localization and extraction.

The observed class imbalance reflects the natural distribution of label layouts encountered during routine diagnostic workflows rather than an artifact of dataset construction. Frequently used lab workflows naturally produced substantially larger numbers of labels than less common or legacy label formats. No artificial balancing strategy was applied because the objective was to preserve the natural distribution of label layouts encountered in routine lab practice.

Fig. 1 shows the distribution of sample counts across groups, highlighting the class imbalance with larger cohorts in G1–G3 and smaller groups in G9–G10.

**Fig. 1:**
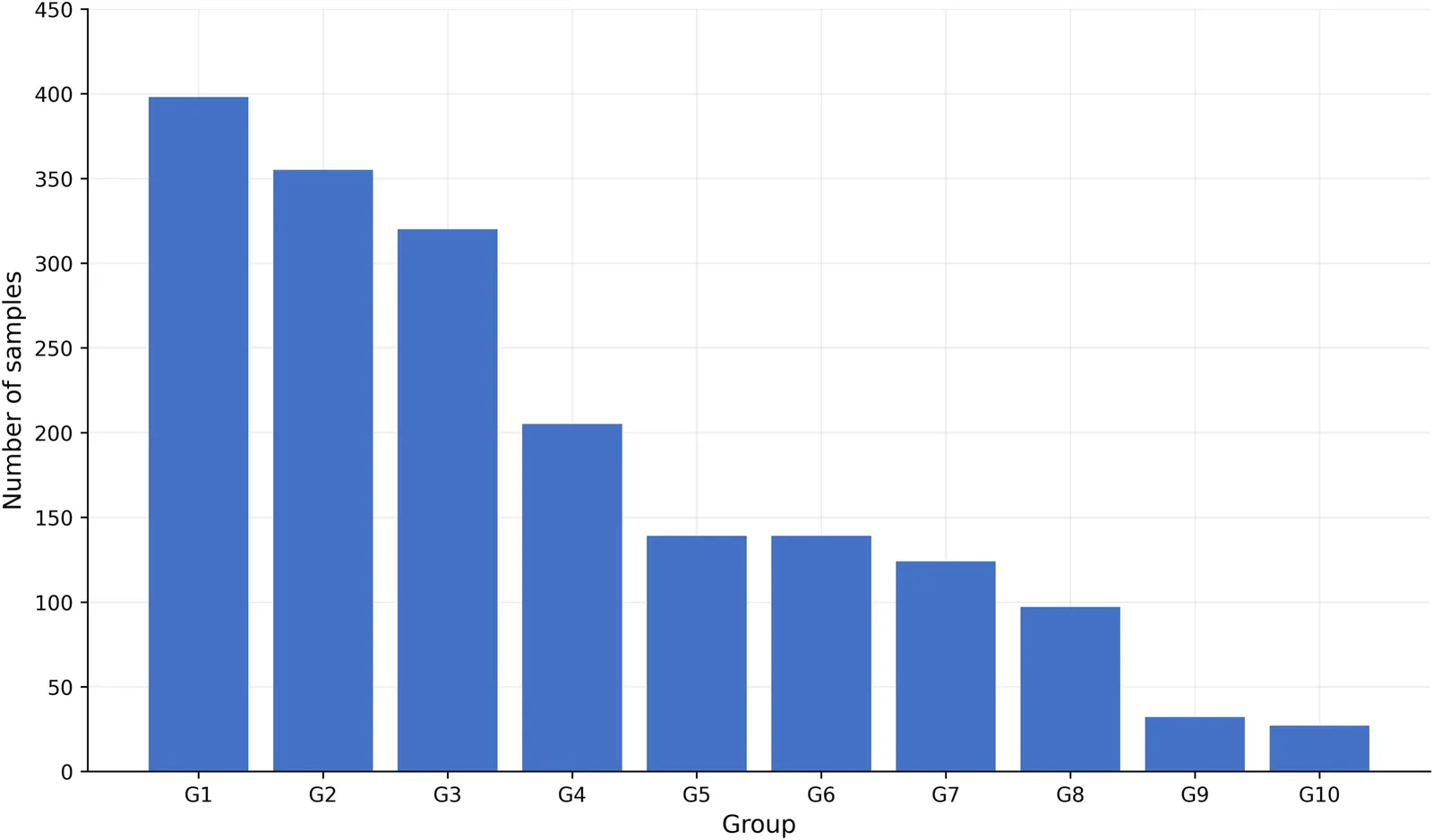
Distribution of sample counts across groups (G1–G10). The figure illustrates the number of slides in each group, ranging from 27 samples in G10 to 398 samples in G1.

Representative examples of the 10 label-layout groups are illustrated in Fig. 2. To preserve patient confidentiality, all displayed labels were reconstructed using fictitious case identifiers while retaining the original spatial arrangement of metadata elements.

**Fig. 2:**
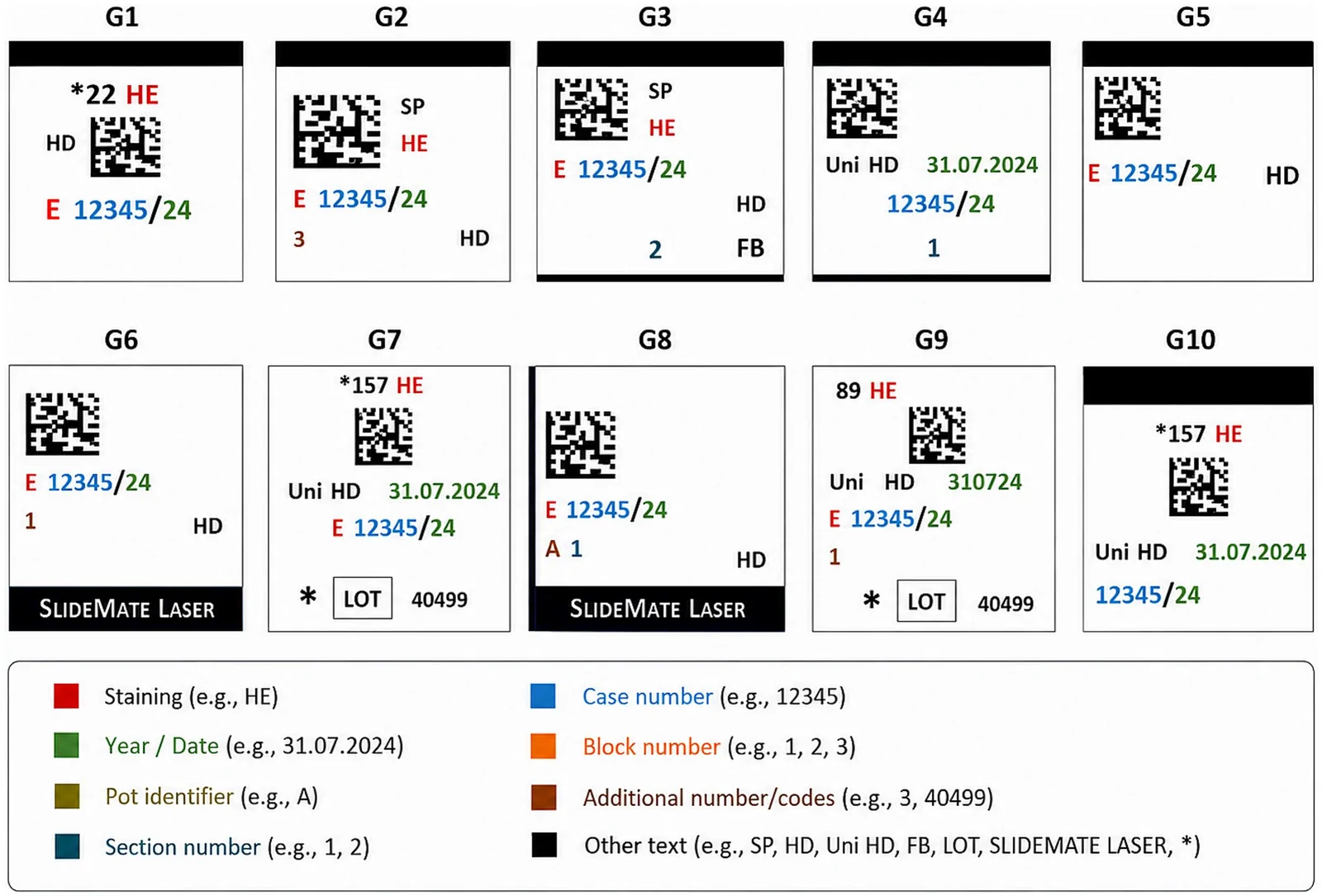
Representative examples of the 10 manually defined label-layout groups (G1–G10) used in WSI-Babel-Shark. Each panel illustrates the characteristic spatial arrangement of metadata fields used for ROI definition and subsequent metadata extraction. Colored text denotes the metadata fields extracted by the pipeline: Lab ID (red), Case number (blue), Year/Date (green), Staining (dark red), Block number (orange), Pot identifier (olive), and Section number (teal). All remaining text shown in black represents layout-specific information, including manufacturer labels, lab abbreviations, lot numbers, auxiliary numeric codes, and other institution-specific printed elements that are not used for SlideID generation. When an alphanumeric code is present (e.g., A1), the alphabetic character represents the Pot identifier, whereas the following numeric character represents the Block number. Stand-alone numeric values appearing separately on the label correspond to the Section number. (For interpretation of the references to color in this figure legend, the reader is referred to the web version of this article.)

The dataset was subsequently divided into training and testing subsets (80/20) using stratified sampling to maintain proportional representation across all groups, and was used exclusively for the development and validation of the label-layout classifier within the known layout domain.

#### Unknown-class dataset for open-set evaluation

To assess the classifier's ability to recognize previously unseen label layouts, we assembled an additional dataset of 61 slide-label images that did not match any of the 10 predefined layout groups. These images included handwritten labels, labels with partially obscured or incomplete metadata, labels without a data matrix code, and legacy label formats that were not represented in the main training dataset. The 61 images were excluded from all training and validation steps and were used exclusively for open-set evaluation to test the classifier's ability to identify and reject novel or non-standard label configurations.

#### Independent dataset for end-to-end and fallback evaluation

To evaluate the WSI-Babel-Shark pipeline in an end-to-end setting under conditions representative of routine diagnostic workflows, we compiled an independent dataset of 270 WSIs scanned on 2 platforms (Aperio and Hamamatsu) and stored in both .svs and .ndpi formats. Each slide contained a verified lab and case identifier, providing reliable ground truth for the assessment of barcode decoding, layout classification, and fallback OCR-based extraction.

In contrast to the dataset used in the earlier WSI-Babel-Fish study, which consisted solely of .ndpi files and followed two predefined label layouts, the present dataset includes slides with six distinct label configurations used in routine diagnostics. These configurations encompass variations in lab prefixes, case identifier formatting, and stain annotation. This diversity provided a more realistic representation of lab variability and served as a robust benchmark for evaluating WSI-Babel-Shark's adaptability and generalization.

#### Cross-dataset compatibility with WSI-Babel-Fish

For comparative analysis and backward-compatibility testing, we reprocessed the dataset from the previously published WSI-Babel-Fish framework. The WSI-Babel-Fish training set was used exclusively to train the label-layout classifier, whereas the WSI-Babel-Fish test set (110 slides) was re-analyzed using WSI-Babel-Shark under identical conditions. This setup ensured strict separation between training and evaluation data and enabled a direct comparison of methodological continuity, reproducibility, and performance between the two systems.

Although both datasets originated from the same institutional archive, they differed significantly in their temporal span and structural composition. The WSI-Babel-Fish dataset contained only slides from 2024, whereas the WSI-Babel-Shark evaluation dataset included material from both 2024 (*n* = 129), 2025 (*n* = 27), and 2026 (*n* = 113). The WSI-Babel-Shark dataset also exhibited greater diversity in case numbering and label design, comprising 76 unique case identifiers compared with 14 in WSI-Babel-Fish. The number of distinct pot identifiers was 7 in WSI-Babel-Shark and 12 in WSI-Babel-Fish, whereas the number of block identifiers decreased from 18 in WSI-Babel-Fish to 9 in WSI-Babel-Shark. Additionally, the WSI-Babel-Shark dataset exhibited substantially greater staining variability, encompassing 60 distinct stain categories compared with 28 in WSI-Babel-Fish. Furthermore, whereas WSI-Babel-Fish comprised two predefined label layouts, the WSI-Babel-Shark dataset contained six distinct label-configuration groups derived from multiple scanner systems.

Collectively, these differences resulted in a more heterogeneous and representative dataset for assessing WSI-Babel-Shark's robustness and generalization across temporal, structural, and staining variations.

### Pipeline architecture and components

#### Overview

The WSI-Babel-Shark framework is a modular, containerized, and YAML-configured workflow for automated extraction, normalization, and consolidation of slide-level metadata from WSIs. Developed in Python, the system integrates decoding of data matrix code, OCR, rule-based parsing, stain recognition, and database registration within a unified and reproducible architecture.

Incoming WSIs are continuously collected from designated monitoring folders and automatically registered for processing. Each slide is screened for prior inclusion in the central registry to prevent duplication and ensure traceable versioning of processed data. After ingestion, label regions are isolated through a vendor-neutral interface capable of accessing embedded image layers within the WSI structure.

When a dedicated label image is not embedded within the whole-slide file, the corresponding macro-overview image is automatically utilized to localize and extract the label region. This fallback ensures reliable metadata acquisition across heterogeneous scanner formats, including those that do not explicitly store label images within their metadata. The extracted label crops are geometrically normalized, rotated to a canonical orientation, and exported as standardized image files that serve as inputs for downstream metadata extraction stages.

A schematic overview of the complete data flow, module interdependencies, and orchestration logic is provided in Fig. 3.

**Fig. 3:**
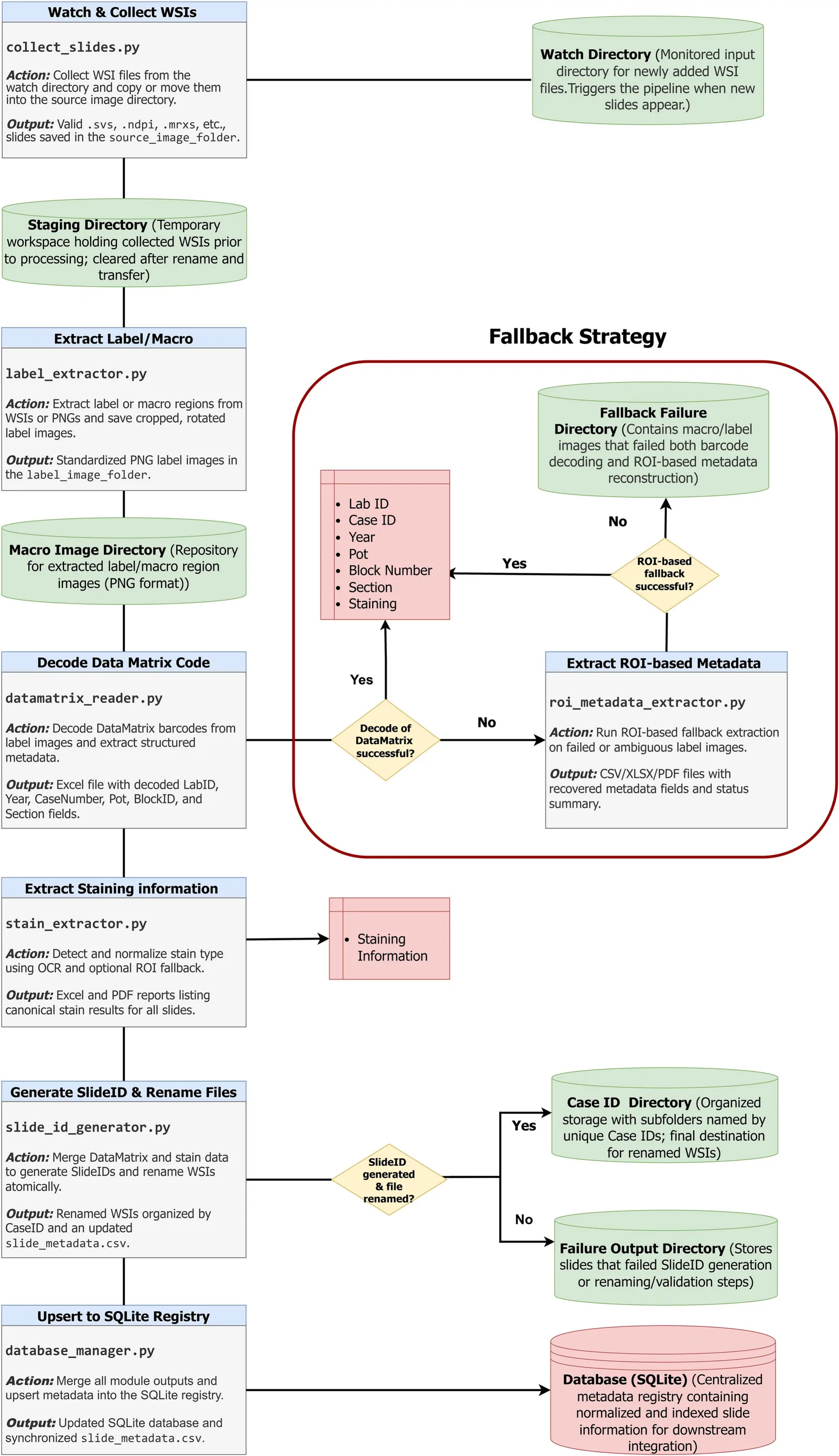
Workflow of the WSI-Babel-Shark main pipeline. The flowchart illustrates the sequential execution of all processing modules coordinated by runner.py under centralized YAML configuration control. The unified command-line interface implemented in cli_main.py manages ROI-based metadata extraction and fallback operations. Together, these modules enable fully automated and reproducible processing of whole-slide images—from initial slide collection and label extraction to data matrix code decoding, stain identification, SlideID generation, and integration of all metadata into the structured SQLite registry.

#### Primary pathway: Data matrix code decoding

The primary extraction route captures metadata directly from two-dimensional (2D) data matrix codes printed on slide labels. Decoding is implemented via the pylibdmtx library^10^ combined with OpenCV-based^11^ image preprocessing and adaptive thresholding, enabling resilience to variation in resolution, illumination, and partial occlusion.

Each data matrix code string follows a structured alphanumeric convention (e.g., E2024001546SA-1-1), where the prefix (“E”) denotes the diagnostic unit, followed by the four-digit year, six-digit case number, and the separator “S” preceding the pot identifier (e.g., “A”). The subsequent numerical fields indicate the block number and section index, respectively.

Each successfully decoded string is parsed using regular-expression templates into structured metadata fields encompassing lab identifier, case number, pot identifier, block number, and section indices. Decoded entries undergo internal consistency validation and are forwarded to the central metadata registry.

This high-fidelity pathway establishes a deterministic linkage between digital slides and lab records while minimizing dependence on text-based recognition methods.

#### Fallback pathway: Classifier-guided ROI and OCR

When data matrix code decoding is not feasible because of missing, damaged, or non-standard codes, the framework activates a classifier-guided fallback procedure that relies on targeted OCR.

A dedicated label-layout classifier integrates convolutional feature embeddings with structural and intensity descriptors to predict the most likely layout category. The predicted layout determines the predefined ROI configuration used for text extraction. ROI definitions were established during system development using manually annotated reference templates created with an in-house annotation utility, ensuring consistent and reproducible localization of key metadata fields across all label types.

Each ROI is subjected to a sequence of image preprocessing operations, including scaling, denoising, and contrast-limited adaptive histogram equalization implemented through OpenCV and NumPy.^12^ OCR inference is subsequently performed using EasyOCR^13^ to capture text within each designated region. The extracted text segments are normalized and processed by a rule-based parsing engine that automatically corrects typographical and segmentation errors.

This fallback pathway provides a reliable mechanism for metadata recovery from heterogeneous labels, including partially obscured, or labels without data matrix code while preserving compatibility with institutional formatting conventions and ensuring high overall robustness of the metadata extraction workflow.

#### Stain extraction and slide identifier

Stain information is not encoded within the data matrix code or any other barcode format in our institution. Instead, it is extracted through OCR applied to the stain-designated region of the slide label. The recognized text tokens are matched against a locally curated stain vocabulary using fuzzy string matching to account for variations in spelling, punctuation, and OCR noise. Matched tokens are subsequently normalized to canonical terminology to ensure consistent representation across slides.

When multiple stain labels are detected, they are automatically merged into standardized composite forms—for example, “PAS&HE” to denote mixed or dual staining procedures. In routine hematoxylin–eosin (H&E) slides used in hematological diagnostics, the stain name is frequently not printed on the label. Consequently, incomplete, degraded, or missing label information can cause the initial OCR result to default to “H&E.” When no valid stain is identified, or when this default behavior occurs, the pipeline triggers an automated ROI- and classifier-guided re-evaluation of the stain region. This secondary analysis increases accuracy by correcting potential misclassifications and detecting compound or non-standard stains that may not have been captured in the initial pass.

Validated metadata from both the data matrix code pathway and the fallback OCR pathway are subsequently integrated with the normalized stain result to construct a standardized slide identifier (SlideID). Each digital slide is then atomically renamed through transactional file operations and reorganized into a hierarchical, case-based directory structure, ensuring complete traceability, reproducibility, and consistent linkage between digital slides and lab records.

#### Database and orchestration

All consolidated metadata are stored in a normalized SQLite registry implemented with the native sqlite3^14^ and pandas^15^ interfaces. Each update operation is performed atomically to prevent partial writes and ensure transactional integrity. In parallel, mirrored CSV summaries are generated to facilitate interoperability with external systems and to support long-term archival. The database schema provides a fully queryable and auditable record of case-level identifiers, stain annotations, and file mappings, enabling transparent traceability across the entire workflow.

The execution of all pipeline components is managed by a dedicated orchestration layer that validates configuration files, resolves inter-module dependencies, and coordinates both batch and continuous-monitoring modes. The orchestration logic adheres to unified coding and documentation standards, including explicit module-level docstrings, structured logging, and comprehensive exception handling. These conventions ensure deterministic reproducibility, fault tolerance, and ease of maintenance across diverse computing environments.

A complete specification of parameter configurations, software dependencies, and workflow blueprints is provided in Supplementary File 1.

### Label layout classifier

A dedicated label layout recognition module was developed to automatically classify the structural type of slide labels and guide ROI selection during fallback metadata extraction. This module enables WSI-Babel-Shark to adapt dynamically to heterogeneous label formats originating from different scanners and institutions. By determining the label layout before OCR, the system automatically selects the appropriate ROI configuration, ensuring that metadata such as lab identifier, case number, pot identifier, block identifier, section number, and stain information are extracted from the correct image regions.

Unlike conventional end-to-end discriminative classifiers, the proposed method follows a prototype-based similarity learning strategy combined with open-set recognition. Instead of directly predicting class labels through a trainable fully connected classification layer, the classifier represents each layout category by a prototype generated from representative training samples. During inference, each incoming label image is compared with all stored prototypes, allowing the classifier not only to recognize known layouts but also to reject previously unseen label structures.

Deep image representations are extracted using a pretrained ResNet-18 convolutional neural network.^16^ The network is employed exclusively as a fixed feature extractor, with the final fully connected classification layer removed. All weights are initialized from the ImageNet pretrained model, and no additional fine-tuning is performed. Each input label image is resized according to the standard ResNet preprocessing pipeline and converted into a normalized 512-dimensional embedding describing the global structural characteristics of the label. This design substantially reduces training requirements while maintaining high generalization across different label appearances.

To complement the deep representation, a handcrafted layout descriptor is extracted from every label image. This descriptor captures structural information that may not be fully represented by the convolutional embedding alone. Specifically, it combines grayscale projection profiles, edge-density distributions, low-frequency Fourier transform coefficients, hue–saturation–value color statistics, hue histograms, and local ink-density measurements. All descriptor vectors are independently L2-normalized before feature fusion. These complementary descriptors improve robustness to differences in typography, printing quality, illumination, and scanner-specific color characteristics.

For every predefined layout group, a prototype representation is generated from the training dataset. Each prototype consists of three complementary prototype representations: (i) the mean ResNet embedding, (ii) the mean handcrafted layout descriptor, and (iii) the normalized average grayscale image representation computed from all training samples belonging to the corresponding layout class. These prototype vectors are stored as compact NumPy index files (.npz), enabling rapid loading during inference without retraining the classifier. Because only prototype representations are retained, the memory footprint remains small while preserving representative information for each layout category.

Classification is performed using a weighted similarity fusion strategy. For every incoming label image, three cosine similarity scores are computed independently by comparing the extracted ResNet embedding, average grayscale representation, and handcrafted layout descriptor with the corresponding prototype of each layout class. The final similarity score is calculated as:

$$S={\mathrm{\alpha S}}_{\mathrm{CNN}}+{\mathrm{\beta S}}_{\mathrm{AVG}}+{\mathrm{\gamma S}}_{\mathrm{Layout}}$$

where $S_{\mathrm{CNN}}$, $S_{\mathrm{AVG}}$, and $S_{\mathrm{Layout}}$ denote the cosine similarities of the deep embedding, average image representation, and handcrafted descriptor, respectively. The weighting coefficients were empirically selected using the validation dataset and fixed to (α = 0.60), (β = 0.20), and (γ = 0.20), assigning greater importance to the deep representation while preserving complementary information provided by the average grayscale representation and handcrafted layout descriptors.

To improve robustness in practical deployment, WSI-Babel-Shark incorporates an adaptive open-set recognition mechanism. Instead of forcing every sample into one of the predefined layout groups, the classifier evaluates both global and class-specific confidence thresholds learned from an independent validation dataset. During threshold estimation, the similarity distributions of correctly classified validation samples are analyzed to derive class-specific acceptance thresholds and confidence margins. During inference, if the highest similarity score does not exceed the corresponding acceptance threshold or fails the confidence-margin criteria, the sample is automatically assigned to the “Unknown” category rather than being forced into an incorrect layout class. This strategy substantially improves reliability when previously unseen label formats are encountered in routine lab practice.

The complete classifier is implemented in Python using PyTorch^17^ for deep feature extraction, OpenCV for image preprocessing, and NumPy for feature normalization and similarity computation. The implementation supports both CPU and GPU execution without modification. Prototype generation, threshold learning, validation, and inference are organized as independent processing stages through a unified configuration-driven command-line interface (CLI), facilitating reproducible deployment and straightforward adaptation to new institutions or additional label layouts. Throughout execution, all predictions, similarity scores, confidence thresholds, and decision outcomes are automatically recorded to ensure full traceability and reproducibility of the classification process.

Finally, by combining deep convolutional representations, handcrafted structural descriptors, prototype-based similarity learning, and adaptive open-set recognition, WSI-Babel-Shark provides a flexible and computationally efficient framework for robust label layout recognition across heterogeneous WSI environments. This design eliminates the need for end-to-end classifier training while providing a compact and reusable feature representation for layout comparison.

### Classifier training and ROI annotation GUI tools

To enhance usability and streamline the creation of customized models and configurations, WSI-Babel-Shark provides a set of graphical user interface (GUI) tools dedicated to classifier training, evaluation, and ROI annotation (Fig. 4). These applications were developed in Python using the CustomTkinter^18^ framework and are designed to simplify the technical processes of classifier construction and label annotation for non-programming users while maintaining full compatibility with the WSI-Babel-Shark pipeline.

**Fig. 4:**
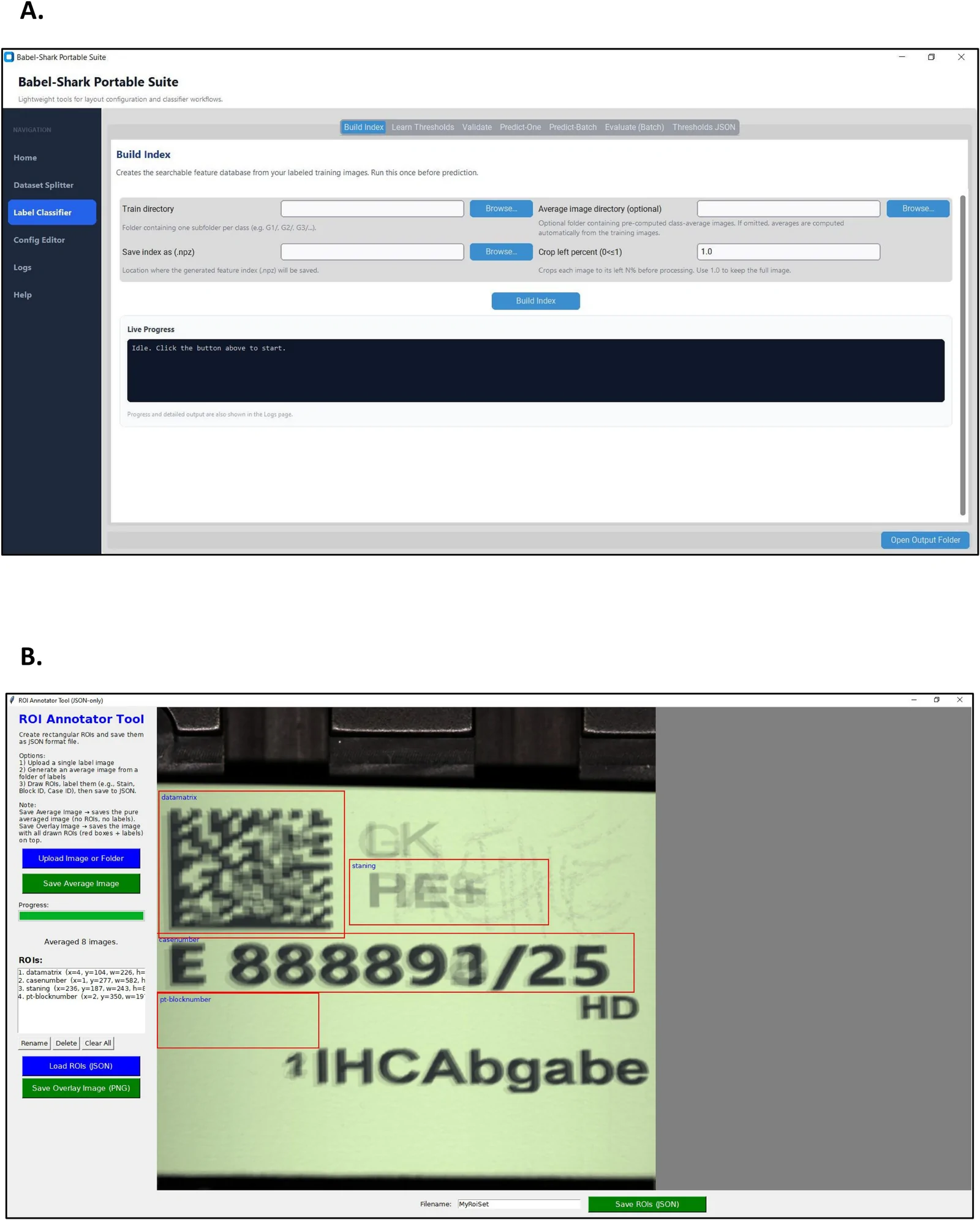
Graphical user interfaces developed for WSI-Babel-Shark. (A) Screenshot of one module of the classifier training application, illustrating the graphical environment for prototype generation, model training, validation, and classifier evaluation. The complete application comprises multiple modules supporting the full classifier development workflow, including dataset management and configuration editing. (B) Screenshot of the stand-alone ROI annotator showing manual delineation of ROIs corresponding to predefined metadata fields on an averaged representative slide-label image. The annotated ROIs are exported as standardized JSON templates, which are automatically loaded following label-layout classification to guide ROI-based metadata localization before OCR during the fallback workflow.

The classifier training GUI provides an interactive environment for building new label layout classifiers through a user-guided workflow that integrates dataset import, feature extraction, prototype generation, and validation. Users can train and evaluate layout classifiers from annotated datasets without command-line operations. Real-time visualization of training progress, class distribution, and accuracy metrics is displayed throughout the process. Upon completion, the system automatically computes confusion matrices, precision-recall metrics, and open-set rejection statistics. Results are exported as structured Excel and JSON reports, and the generated prototype indices are stored as compact NumPy archives (.npz) for direct deployment within the WSI-Babel-Shark pipeline. The classifier training interface is illustrated in Fig. 4A, demonstrating the interactive workflow for dataset preparation, classifier training, prototype generation, and model evaluation.

Beyond model training, the GUI includes dedicated interfaces for configuration and dataset management. The configuration editor provides an interactive environment for creating and modifying YAML^19^ and JSON files with automatic syntax validation, parameter range checking, and path normalization across operating systems. The dataset manager supports controlled splitting of image data into training, validation, and testing subsets using stratified sampling with fixed random seeds to ensure reproducibility. All operations generate timestamped logs and human-readable documentation for transparency and traceability.

Complementing the classifier GUI, WSI-Babel-Shark incorporates a stand-alone ROI annotation tool that replaces the dependency on ImageJ present in the earlier WSI-Babel-Fish framework. For each manually defined layout group, a representative average label image was generated by pixel-wise averaging all label images belonging to that group. These average images served as layout-specific templates for manual ROI definition. Using the ROI annotator application, rectangular ROIs corresponding to metadata fields, including the data matrix code, lab identifier, case identifier, block identifier, stain information, and other relevant printed elements, were manually delineated and assigned semantic labels. The ROI coordinates are subsequently exported as standardized JSON configuration files. The ROI annotation interface is shown in Fig. 4B, illustrating the interactive definition of metadata regions and the generation of standardized JSON-based ROI templates for automated ROI localization before OCR.

During metadata extraction, the label layout classifier first determines the corresponding layout group, after which the associated JSON ROI template is automatically loaded to localize the metadata regions before OCR, enabling robust extraction despite substantial variability in label layout. The tool employs OpenCV and Pillow^20^ for image rendering, NumPy for numerical operations, and jsonschema^21^ for configuration validation.

Both the classifier training GUI and the ROI annotation tool were designed as independent, stand-alone applications operating outside the core WSI-Babel-Shark pipeline but producing standardized outputs fully compatible with it. This modular design allows classifier development and ROI definition to be performed independently, facilitating flexible integration, version control, and reproducibility across different deployment environments.

In addition to its role within WSI-Babel-Shark, the ROI annotation tool was released as an independent open-source utility in a separate public repository to encourage reuse and adaptation in other biomedical imaging and computer-vision projects. Its modular design and standardized JSON export format make it suitable as a lightweight, general-purpose annotation interface for tasks beyond digital pathology, including document analysis and microscopy-based image labeling. By providing accessible graphical interfaces for both classifier training and ROI creation, WSI-Babel-Shark bridges the gap between advanced computational functionality and practical usability in real-world lab workflows.

### Evaluation design

The evaluation strategy of WSI-Babel-Shark was designed to comprehensively assess both the performance of the label-layout classifier and the robustness of the complete metadata extraction pipeline under realistic diagnostic conditions. All experiments were performed on datasets that were entirely independent of those used for classifier training and development, ensuring unbiased and reproducible results.

#### Classifier evaluation

The label-layout classifier was evaluated under both closed- and open-set conditions using the datasets described in the “Datasets” subsection of Materials and methods. Closed-set evaluation was performed on the 20% stratified test split of the 1836-label dataset, whereas open-set performance was assessed using 61 unseen label images representing handwritten or slides without data matrix code. Quantitative performance was measured using accuracy, precision, recall, F1-score, confusion matrices, and open-set rejection rates. These metrics captured both the classifier's ability to correctly recognize known label structures and its capacity to reject previously unseen configurations.

#### Pipeline and fallback evaluation

To evaluate WSI-Babel-Shark under conditions representative of routine diagnostic workflows, an independent set of WSIs was selected from multiple scanner platforms to capture real-world variability in label layouts and file structures. Each slide label contained both a verified 2D data matrix code and human-readable printed text encoding lab , case, and specimen identifiers, ensuring reliable ground-truth metadata for validation across both data matrix code- and OCR-based extraction pathways.

The evaluation was performed in two complementary modes designed to characterize standard and fallback operation. In the standard mode, the complete WSI-Babel-Shark pipeline was executed end-to-end, beginning with data matrix code decoding, followed by ROI-based OCR, metadata parsing, and database registration. This configuration represented typical diagnostic conditions in which data matrix code information was available and readable.

In the fallback mode, data matrix code decoding was intentionally disabled to activate the OCR-based extraction pathway. In this configuration, metadata retrieval relied entirely on the label-layout classifier, predefined ROI configuration, OCR recognition, and rule-based parsing. This mode simulated practical failure scenarios including missing, damaged, or occluded data matrix codes, allowing direct assessment of the fallback mechanism's contribution to the pipeline's overall reliability.

For each slide, metadata obtained through the fallback pathway were directly compared with the ground-truth metadata decoded from the corresponding data matrix codes. This comparison enabled quantitative estimation of field-specific accuracy and concordance across identifiers including case number, block ID, section number, and staining information. Slides with partial decoded of data matrix code were further analyzed to delineate the operational boundaries and robustness of the fallback module under heterogeneous label conditions.

#### Cross-framework reproducibility

To verify backward compatibility and ensure methodological continuity, WSI-Babel-Shark was benchmarked against the previously published WSI-Babel-Fish framework using datasets from the original study. The WSI-Babel-Fish training subset was employed exclusively to retrain and adapt the WSI-Babel-Shark label-layout classifier, ensuring full compatibility of feature representations and ROI configurations between the two systems. The corresponding WSI-Babel-Fish test dataset was then reprocessed through the complete WSI-Babel-Shark pipeline, including its ROI-based fallback module, under identical preprocessing and evaluation parameters. This controlled benchmarking design enabled a direct, framework-level assessment of reproducibility and methodological consistency across versions.

### Statistical and implementation details

All experiments were conducted in a controlled Linux environment using Python 3.11 as the primary programming language.

The WSI-Babel-Shark framework was implemented as a fully modular and containerized system to ensure reproducibility, maintainability, and platform independence.

A dedicated Docker image^22^ was developed to encapsulate all dependencies, enabling consistent deployment across heterogeneous computational environments.

Core image-handling and preprocessing operations were implemented using OpenSlide, OpenCV, and Pillow, whereas numerical computation and data manipulation relied on NumPy and pandas.

OCR in the fallback extraction pathway was performed using EasyOCR, fully integrated within the ROI-based workflow.

Deep learning components, including the label-layout classifier, were developed using PyTorch and scikit-learn^23^ backends.

Configuration management was handled through YAML files, and metadata storage utilized SQLite3 for lightweight, file-based database integration.

Each module was designed as an independent CLI supporting standardized argument parsing, structured logging, and atomic file writing to prevent data corruption during batch operations.

A central orchestration layer managed task sequencing, dependency validation, and exception handling, while maintaining reproducible log files for every execution.

Logging adhered to a unified format with timestamped INFO, WARNING, and ERROR entries to ensure traceability and auditability.

Statistical analyses of performance metrics were carried out using NumPy and SciPy libraries.

Evaluation metrics included classification accuracy, precision, recall, and F1-score for the label-layout classifier, as well as field-level concordance rates for pipeline- and fallback-derived metadata.

Comparative analyses between data matrix code- and OCR-based outputs employed pairwise string matching and regular-expression–based normalization to ensure consistent identifier comparison.

### Code and data availability

The complete WSI-Babel-Shark framework, including the source code, YAML configuration templates, and documentation, is publicly available under an open-source license through the project's GitHub repository (https://github.com/cpheidelberg/WSI-Babel-Shark).

Two companion tools are provided through independent GitHub repositories: (i) the ROI annotator (https://github.com/cpheidelberg/ROI-Annotator), an interactive application for creating and editing layout-specific ROIs; and (ii) the Label-Layout-Classifier GUI (https://github.com/cpheidelberg/label-layout-classifier), which provides a graphical interface for classifier training, visual inspection, and manual verification of predicted label layouts. Both companion repositories are referenced in the main WSI-Babel-Shark repository to facilitate access and use alongside the core framework.

To support reproducible deployment across different computing environments, a pre-built Docker image is available via Docker Hub (https://hub.docker.com/r/bioinfo2026/wsi-babel-shark).

In addition, a small synthetic test dataset comprising 22 label-only images containing artificial, non-patient metadata is publicly available through heiDATA, the institutional research data repository of Heidelberg University (https://doi.org/10.11588/data/ZBS9RS). This dataset is intended exclusively for installation testing and basic pipeline validation and does not contain real WSIs or any patient-identifiable information.

## Results

### Classifier performance

The label-layout classifier showed strong performance in assigning slides to the correct layout group. On the closed-set test set, which included 20% of the 1836 manually annotated samples, the model achieved an overall accuracy of 97.41%. Class-wise precision, recall, and F1-scores are summarized in Table 1, demonstrating consistently high performance across the 10 predefined layout groups, with macro-averaged precision and recall values of 0.98 and 0.97, respectively.

**Table 1:** Performance metrics for the label-layout classifier.

Class	Precision	Recall	F1-score	Support
G1	1.000	0.960	0.980	79
G2	1.000	1.000	1.000	71
G3	0.950	0.940	0.940	64
G4	1.000	1.000	1.000	41
G5	1.000	0.930	0.960	27
G6	1.000	1.000	1.000	27
G7	1.000	1.000	1.000	24
G8	0.890	0.890	0.890	19
G9	1.000	1.000	1.000	6
G10	1.000	1.000	1.000	5
Unknown	0.910	1.000	0.950	61
Overall accuracy	–	–	0.974	424
Macro Avg	0.980	0.970	0.980	424
Weighted Avg	0.975	0.974	0.974	424

Precision, recall, F1-score, and support are provided for each predefined layout group (G1–G10) and for the open-set “Unknown” category. Macro- and weighted-average values summarize overall classifier performance on the 424-sample test set.

These findings further support the robustness of the proposed label-layout classifier across heterogeneous label configurations. This robustness is evidenced by consistently high classification performance despite substantial class imbalance, accurate rejection of previously unseen layouts through open-set evaluation, and the observation that the few remaining misclassifications were confined to structurally similar label categories.

The confusion matrix in Fig. 5 shows that errors were concentrated in a small number of cases and occurred primarily between layout groups with very similar structures. These misclassifications were mainly related to minor shifts in the position of the barcode or text fields and did not indicate systematic confusion between unrelated groups.

**Fig. 5:**
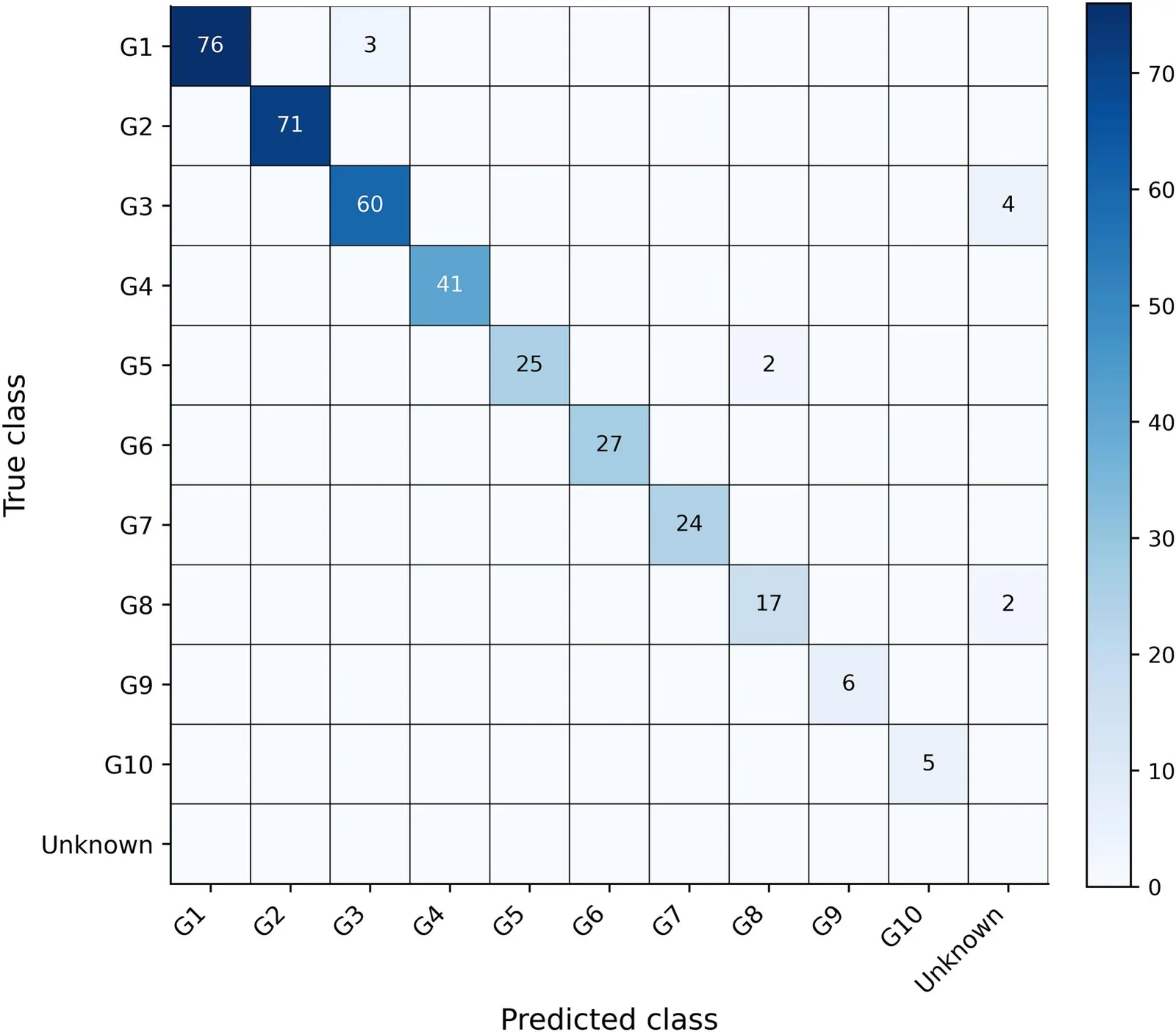
Confusion matrix of the label-layout classifier. The figure summarizes the classifier's performance on an independent 424-sample test set across all predefined layout groups (G1–G10) and the open-set “Unknown” category. Strong diagonal dominance indicates high agreement between predicted and true classes, whereas the few off-diagonal entries represent misclassifications between visually similar label layouts.

Open-set performance was evaluated on 61 previously unseen labels, including handwritten, heterogeneous, or non-standard layouts. The classifier identified all of these samples as out-of-distribution, yielding a recall of 1.00 and a precision of 0.91 for the “Unknown” class, resulting in a F1-score of 0.95. These results demonstrate that the model distinguishes known institutional layouts from non-standard formats with high reliability.

### Pipeline performance

The complete WSI-Babel-Shark pipeline was evaluated on 270 WSIs representing 5 predefined label-layout groups acquired from 2 scanner platforms. Label regions were successfully detected and extracted from all 270 WSIs, generating standardized label crops for downstream processing. Direct data matrix code decoding reconstructed the required metadata for 236 slides (87.4%). The remaining 34 slides (12.6%), for which the data matrix codes were damaged, partially occluded, or unreadable, were processed using the classifier-guided ROI-based fallback extraction pathway. Within the fallback workflow, the label layout was first classified, followed by automatic loading of the corresponding ROI template and OCR-based metadata extraction. Metadata were extracted for 31 of the 34 fallback cases, whereas metadata extraction failed for the remaining 3 slides because label-layout classification and/or OCR-based metadata extraction could not recover the required metadata. These three slides were excluded from the subsequent quantitative analysis. Consequently, 267 slides were included in the final performance evaluation.

Following metadata extraction, stain information was extracted from all 267 evaluable label images using the OCR-based stain extraction module. The extracted stain information was subsequently combined with the recovered metadata to generate the final SlideID and automatically rename the corresponding WSIs.

According to the predefined evaluation criterion, a SlideID was considered correct only when all required metadata fields were extracted accurately. Based on this criterion, 258 of the 267 evaluable slides (96.6%) were reconstructed without error, whereas the remaining 9 slides (3.4%) contained one or more metadata extraction errors.

Field-level analysis demonstrated high extraction accuracy across the individual metadata components. The case number and year were correctly extracted for all 267 evaluable slides. Three extraction errors were observed for the lab identifier, three for the pot identifier, three for the block identifier, and three for the stain annotation. Because multiple metadata fields were incorrect in some slides, these errors were distributed across nine slides rather than representing independent failure cases. The observed extraction errors were associated with degraded label quality, including low print contrast, partial label damage, glare artifacts, handwritten markings, mixed-format stain annotations, and OCR confusion between visually similar individual characters or digits during character-level recognition.

To further evaluate the accuracy of the OCR-based metadata extraction independently of the routine pipeline, the 236 slides with successfully decoded data matrix codes were additionally processed through the classifier-guided ROI-based OCR pathway. The OCR-derived metadata were compared with the corresponding data matrix-decoded metadata on a field-by-field basis. Complete agreement across all evaluated metadata fields was observed for 230 of 236 slides (97.5%). Field-level concordance reached 100.0% for the lab identifier and year, 99.6% for the case number, 99.2% for the pot identifier, and 98.3% for the block identifier.

### Cross-framework comparison

When evaluated on the legacy dataset previously used in the WSI-Babel-Fish study, WSI-Babel-Shark maintained a comparable level of accuracy across all metadata fields.

As summarized in Table 2, OCR-based retrieval achieved near-perfect identification rates for lab prefix, case number, year suffix, block number, and staining information in both training and testing subsets.

**Table 2:** Results of the ROI-based metadata extraction module.

Label information	Correctly identified (Accuracy)	
	WSI-Babel-Fish dataset	WSI-Babel-Shark dataset
Prefix/lab ID	110/110 (1.000)	264/267 (0.989)
Case number	110/110 (1.000)	267/267 (1.000)
Suffix/year	110/110 (1.000)	267/267 (1.000)
Pot identifier	107/110 (0.973)	264/267 (0.989)
Block number	108/110 (0.982)	264/267 (0.989)
Staining	109/110 (0.991)	264/267 (0.989)
Entire information set	105/110 (0.955)	258/267 (0.966)

The module was evaluated on 2 independent datasets comprising 110 (WSI-Babel-Fish) and 270 (WSI-Babel-Shark) slide labels. Three WSI-Babel-Shark labels failed the metadata extraction workflow owing to severe label degradation and were excluded from field-level accuracy analysis, resulting in 267 evaluable slide labels. Individual metadata fields (Prefix/Lab ID, Case number, Suffix/Year, Pot identifier, Block number, and Staining) and the complete information set per slide were subsequently evaluated.

Field-level accuracies remained consistent with those reported for WSI-Babel-Fish, indicating that the new framework preserved the reliability of the original implementation while introducing a more modular and reproducible architecture.

Minor variations were primarily observed in lab identifiers, pot identifiers, block identifiers, and stain annotations and were mainly attributable to label artifacts such as glare, partial occlusion, or data matrix code degradation rather than model misclassification.

## Discussion

### Discussion

The WSI-Babel-Shark framework represents a substantial improvement over its predecessor, WSI-Babel-Fish,^4^ in the automated extraction of slide-level metadata from WSIs. Building on the conceptual foundation established by Babel Fish, it evolves a prototype into a robust and reproducible system capable of operating across heterogeneous lab environments. Whereas the original WSI-Babel-Fish system demonstrated the feasibility of OCR-based metadata retrieval within a single institutional setting, WSI-Babel-Shark extends this approach through deep-learning–based layout recognition, open-set identification of previously unseen label types, and a classifier-guided fallback mechanism that supports reliable metadata recovery even when data matrix code information is incomplete or unavailable. In contrast to Babel Fish, which extracted only predefined metadata fields, WSI-Babel-Shark processes all extractable label information and applies intentional trade-offs to maximize recovery across diverse formats. The principal methodological and architectural differences between WSI-Babel-Fish and the proposed WSI-Babel-Shark framework are summarized in Table 3.

**Table 3:** Comparison of the methodological and architectural characteristics of WSI-Babel-Fish and the proposed WSI-Babel-Shark framework.

Component	WSI-Babel-Fish	WSI-Babel-Shark
Label layout handling	Fixed label layouts	Prototype-based label-layout classifier with automatic layout recognition
Previously unseen layouts	Not supported	Open-set recognition enables rejection of unknown layouts
ROI localization	Manually defined ROI selection	Automatic loading of layout-specific JSON ROI templates after layout classification
ROI annotation	External ImageJ software	Dedicated ROI annotator GUI integrated into the workflow
Metadata extraction strategy	OCR-based extraction from predefined ROIs	Hierarchical extraction combining data matrix decoding, classifier-guided ROI localization, OCR, and rule-based parsing
Barcode redundancy	Not available	Automatic data matrix decoding with OCR fallback
Metadata validation	Limited	Rule-based validation and standardized parsing
Configuration	Manual configuration	Centralized YAML- and JSON-based configuration
Deployment	Prototype implementation	Docker-containerized, modular, reproducible pipeline
Software architecture	Single-purpose research prototype	Modular architecture with independent components for classification, ROI localization, OCR, parsing, and reporting
Extensibility	Limited adaptation to new layouts	New layouts can be incorporated by generating additional prototypes and ROI templates without retraining the entire classifier

WSI-Babel-Shark extends the previous framework through automated layout recognition, open-set classification, classifier-guided ROI localization, standardized ROI annotation, modular software architecture, and containerized deployment, thereby improving adaptability, reproducibility, and scalability across heterogeneous label layouts.

The experimental results confirm that WSI-Babel-Shark performs reliably under a wide range of diagnostic conditions and maintains consistent field-level accuracy across label formats and the two scanner platforms included in this study. The high end-to-end success rate observed in heterogeneous datasets demonstrates the stability of the pipeline and its capability to handle both structured and irregular label layouts. The robustness of the proposed label-layout classifier is further supported by its high classification performance across 10 heterogeneous layout groups despite substantial class imbalance, reliable recognition of previously unseen layouts through the open-set evaluation, and the observation that the few remaining misclassifications occurred only between structurally similar label categories. Importantly, none of the observed errors affected case identification in the present dataset. All discrepancies were limited to block identifiers and stain labels, and these can be corrected manually with minimal effort, preventing any risk of mixing up cases. Minor inconsistencies were primarily attributable to physical artifacts on the label, such as low print contrast, misaligned edges, or partial trimming, rather than model misclassification. These findings highlight that label print quality and scanning conditions remain key determinants of metadata extraction accuracy.

Beyond numerical performance, WSI-Babel-Shark introduces several conceptual and architectural advances that broaden its utility. The classifier-guided fallback strategy dynamically adapts ROI extraction based on the predicted label layout, whereas the open-set module prevents incorrect assignments to known categories. The introduction of GUIs for classifier training and ROI annotation removes reliance on third-party software and enables customization by users without programming expertise. Containerized deployment ensures reproducible execution across computing environments, and an unified YAML-based configuration facilitates transparent parameter management and supports cross-institutional deployment. Together, these features establish WSI-Babel-Shark as an extensible foundation for automated slide-level metadata extraction in digital pathology.

Unlike radiology, where standardized image formats and metadata models have become widely established through DICOM, digital pathology still comprises a heterogeneous ecosystem of scanner vendors, proprietary image formats, and institution-specific slide-label conventions.^7^ Although ongoing standardization efforts are expected to improve interoperability in the future, widespread adoption remains incomplete across many research and diagnostic environments.^6^ Rather than relying on the emergence of a universal labeling standard, WSI-Babel-Shark was designed to operate within this existing heterogeneous landscape by providing layout-aware metadata extraction that can be adapted to diverse label configurations. This pragmatic strategy reduces dependence on uniform label designs and facilitates interoperability across current lab workflows without requiring changes to existing slide-generation processes.

Although WSI-Babel-Shark demonstrates strong robustness and adaptability, several components could be further improved. The fallback OCR pathway remains sensitive to glare, faint printing, and handwritten annotations, which can introduce minor inconsistencies in text recognition. Slides for which reliable metadata reconstruction cannot be achieved are automatically flagged for manual review instead of being assigned potentially incorrect metadata, thereby providing a fail-safe quality assurance mechanism within the current end-to-end pipeline. These issues could be reduced through additional image-normalization steps, adaptive thresholding, or the integration of small handwriting-recognition modules. The rule-based parsing logic, while effective under current institutional naming conventions, may require adjustment when applied in labs that use different identifier formats; developing configurable parsing templates or semi-automatic schema-learning tools would support easier adaptation. In addition, although the open-set classifier reliably detects new label layouts, incorporating domain-adaptation techniques, incremental learning, and periodic self-supervised updates could strengthen its generalization across institutions, scanners, and evolving label designs.

### Strengths and limitations

The proposed WSI-Babel-Shark framework offers several methodological and practical advantages that distinguish it from conventional slide-label processing workflows. A principal strength of the framework is its modular software architecture, which separates label layout classification, ROI localization, OCR, metadata parsing, and configuration management into independent components. This design simplifies maintenance, facilitates future extension, and allows individual modules to be updated or replaced without requiring modifications to the entire pipeline. This modular organization also enables institution-specific adaptation by allowing individual components to evolve independently as lab workflows, scanner technologies, or label formats change over time.

Furthermore, the prototype-based layout classifier enables straightforward incorporation of newly encountered label layouts by generating additional prototypes and corresponding ROI templates rather than retraining a complete deep-learning model. The dedicated ROI annotator application further improves reproducibility by providing a standardized workflow for generating layout-specific JSON-based ROI templates, whereas the graphical configuration tools reduce deployment complexity for users without extensive programming experience.

Several limitations should nevertheless be acknowledged. First, although the software architecture was intentionally designed to support heterogeneous scanner vendors and diverse label formats, the current experimental evaluation was performed using label layouts originating from only two scanner platforms and the label formats routinely encountered at our institution. Consequently, additional validation on datasets acquired from other scanner vendors, lab workflows, and institutions is required to further evaluate the generalizability of the proposed framework. Second, the current implementation requires the one-time creation of layout-specific ROI templates whenever a previously unseen label layout is introduced. Although this process is supported by the dedicated ROI annotator application and does not require modification of the underlying algorithms, it still involves manual user interaction during the initial configuration phase. Third, the present implementation does not yet provide direct integration with LIS or PACS. Accordingly, integration with institutional information systems was not evaluated in the present study. The extracted metadata are exported in standardized formats suitable for downstream processing, and seamless bidirectional communication with institutional information systems remains an important direction for future development. Finally, as with all OCR-based document analysis systems, overall performance remains dependent on image quality, label printing conditions, and the readability of printed text, factors that lie largely outside the control of the software itself.

### Future directions

Future directions for WSI-Babel-Shark include the integration of handwriting-recognition models, adaptive stain classifiers, and multimodal strategies that combine textual and visual features for more comprehensive metadata validation. Additional opportunities include comparing section morphology to detect potential slide mix-ups, aligning section shape with block identifiers, and automatically verifying stain appearance against the printed label annotation. Integration with LIS and PACS through standardized interoperability frameworks, including DICOM-WSI, may further support translation into clinical workflows.

## Conclusion

WSI-Babel-Shark provides an experimentally validated, robust, and extensible solution for automated slide-level metadata extraction by combining layout-aware deep learning, prototype-based open-set recognition, and a classifier-guided fallback strategy within a reproducible, modular architecture. Its broader coverage of heterogeneous label formats and high end-to-end reliability demonstrate meaningful improvements over WSI-Babel-Fish. Whereas minor configuration adjustments may be required across institutions, the framework offers a practical foundation for metadata harmonization in digital pathology. Continued advances in domain adaptation, handwriting interpretation, and stain-label validation are expected to further enhance its generalization and support clinical integration.

## Consent for publication

Not applicable. No identifiable human data are included.

## Ethics approval and consent to participate

All slide-label images used in this study originated from routine diagnostic workflows at the Institute of Pathology Heidelberg. No patient data have been used. In accordance with institutional and national regulations, the use of fully anonymized retrospective material for methodological development does not require formal ethics committee approval.

## Declaration of generative AI and AI-assisted technologies in the writing process

During the preparation of this work, the authors used ChatGPT (OpenAI) to improve clarity, structure, and readability. All AI-generated suggestions were reviewed, edited, and verified by the authors, who take full responsibility for the content of the published article.

## Funding

No funding was received for this study.

## Declaration of competing interest

The authors declare no known competing financial interests or personal relationships that could have influenced the work reported in this article.

## Data Availability

The publicly shareable subset of slide-label images, configuration templates, and example outputs used in this study is available in the heiDATA repository under the persistent identifier: https://doi.org/10.11588/data/ZBS9RS Additional anonymized datasets used for internal validation are available from the corresponding author upon reasonable request and in compliance with institutional data-protection policies. Code availability The complete source code for the WSI-Babel-Shark framework is available at: https://github.com/cpheidelberg/WSI-Babel-Shark A corresponding containerized implementation is available on Docker Hub: https://hub.docker.com/r/bioinfo2026/wsi-babel-shark All code versions used for generating the results in this manuscript are archived within the repository.

## Appendix A Supplementary data

Supplementary material 1 *WSI-Babel-Shark Technical Blueprint* accompanies this article and provides detailed descriptions of the pipeline modules, configuration parameters, and workflow components. Supplementary materials are provided exactly as submitted and have not been typeset.
