# Supplementary material for: WSI-Babel-Shark: A modular pipeline for robust metadata extraction from heterogeneous whole slide image labels in digital pathology
Source: J Pathol Inform. 2026 Sep 24;23:100722. doi: 10.1016/j.jpi.2026.100722 (PMC13721096; doi:10.1016/j.jpi.2026.100722)
Supplement: Supplementary file 1 [file mmc1.pdf]

WSI-Babel-Shark — Technical Blueprint

Supplementary File 1

Corresponding author: Shahram Aliyari
Section Computational Pathology Heidelberg, Institute of Pathology Heidelberg, University Hospital Heidelberg, University of Heidelberg, Heidelberg, Germany.

Date: December 2025

1. Overview & Purpose

WSI-Babel-Shark is a modular, reproducible framework for automated extraction, normalization, and consolidation of slide-level metadata from whole-slide images (WSIs). The system integrates barcode decoding, classifier-guided region-of-interest (ROI) extraction, OCR-based fallback parsing, stain recognition, and database registration into a unified pipeline.

As the successor to *WSI-BabelFish*, Babel-Shark expands beyond single-layout OCR workflows by introducing layout-aware metadata extraction, open-set rejection, cross-scanner compatibility, deterministic logging, and full containerization. The framework is designed for institutional archives, research datasets, and large digital-pathology infrastructures that require standardized, scalable metadata harmonization.

2. System Architecture

The architecture consists of independent Python modules connected through a uniform Command-Line Interface (CLI) layer. All components follow consistent input/output semantics, atomic file operations, and YAML configuration control. Modules can be executed individually or orchestrated through the pipeline runner.

Module	Function	Description
collect_slides.py	Slide ingestion	Monitors input directories, copies/moves WSIs, de-duplicates entries.
label_extractor.py	Label isolation	Extracts label crops using OpenSlide or macro-image fallback.
datamatrix_reader.py	Barcode decoding	Decodes DataMatrix codes and parses structured identifiers.
roi_metadata_extractor.py	ROI-OCR fallback	Applies classifier-guided ROI extraction and OCR-based parsing.
stain_extractor.py	Stain identification	Performs OCR + vocabulary normalization and verification.
slide_id_generator.py	SlideID synthesis	Merges metadata, normalizes IDs, and renames WSIs atomically.
database_manager.py	SQLite registry	Stores normalized metadata and provenance.

runner.py	Pipeline orchestration	Coordinates module execution, fallback logic, logging, and errors.
cli_main.py	Unified CLI	Provides standard subcommands (run, validate, version).

3. Pipeline Data Flow

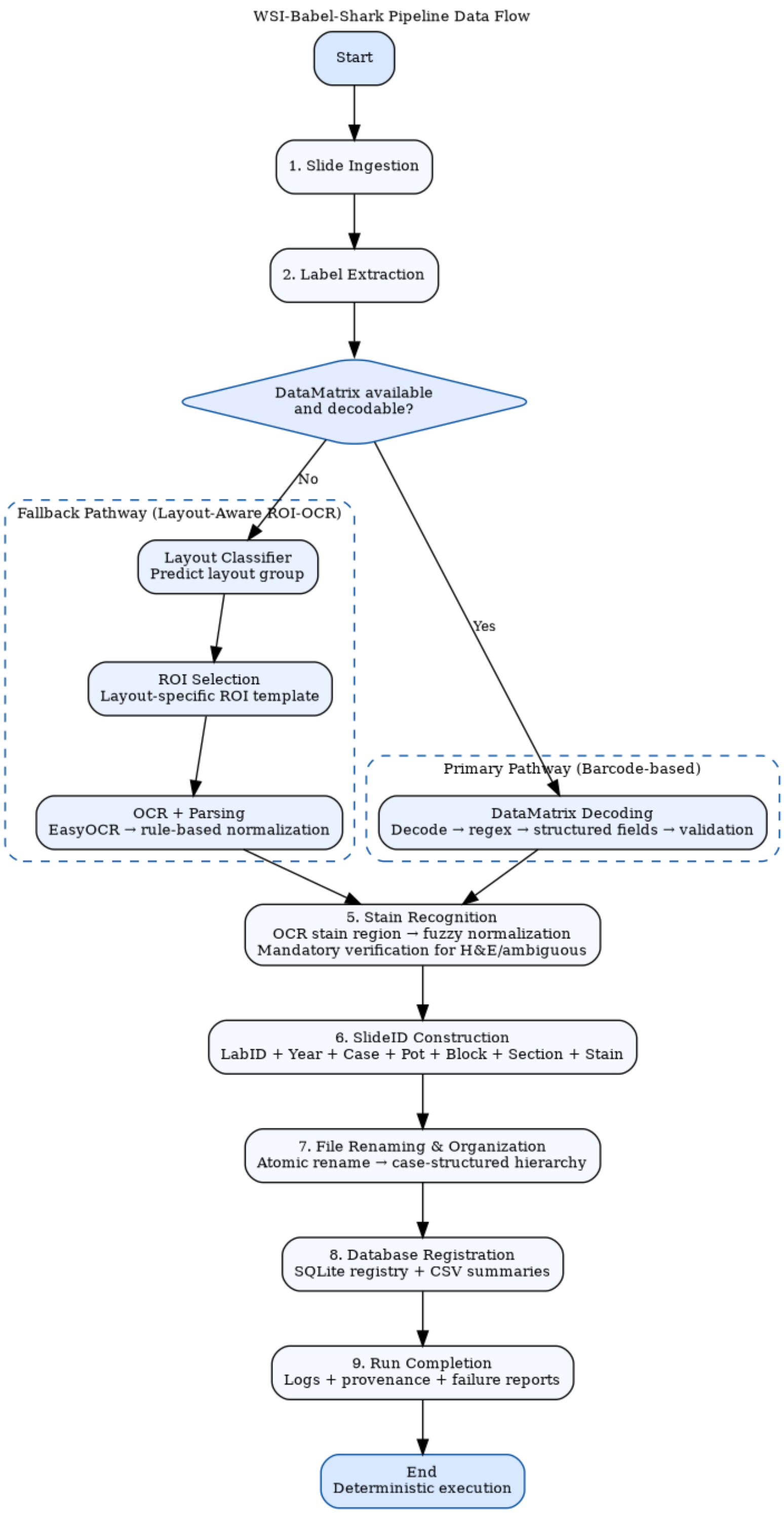

4. Input / Output Specifications

4.1 Inputs

Type	Format	Description
Whole-slide images	.svs, .ndpi, .scn, .mrxs, .bif, .tif	Vendor-neutral via OpenSlide.
Label crops	.png, .jpg	Automatically extracted label regions.
Configuration	.yaml, .json	ROI sets, layout model params, pipeline settings.

4.2 Outputs

Type	Format	Description
Metadata tables	.csv, .xlsx	Structured metadata and per-field extraction results.
Reports	.pdf	Stain summaries and visual validation.
Registry	.db (SQLite)	Normalized metadata and provenance.
Logs	Plain text	Module-level trace and fallback logs.

5. Configuration Parameters

Key parameters from the YAML interface include:

Parameter	Purpose
folder_to_watch	Continuous ingestion directory for WSIs.
source_image_folder	Internal staging area.
output_base_folder	Root directory for pipeline outputs.
operation_mode	Copy or move slides (copy/move).
wsi_types	Allowed extensions.
roi_set_file	ROI configuration JSON.
layout_model.params	Prototype-based layout classifier settings.
stain_dictionary_json	Normalized stain vocabulary.
sqlite_db_path	Path for metadata registry.

6. Error Handling & Fallback Logic

WSI-Babel-Shark uses a tiered fallback strategy:

1. Barcode decoding failure → ROI-OCR fallback
2. Unknown layout prediction → fixed generic ROIset

3. Ambiguous or missing stains → mandatory ROI re-evaluation
4. Persistent parsing failures → slide moved to /failed/ with logs

All error states are fully logged and preserved for reproducibility.

7. Logging & Provenance

Each pipeline run generates:

- A timestamped .logs directory
- Per-module trace files
- Extraction reports and warnings
- Run ID, version, configuration snapshot
- SQLite metadata with insertion timestamps

The logging framework is consistent across modules and ensures full traceability for batch and watch-mode operations.

8. Database Schema (SQLite)

Column	Description
id	Autoincrement key
run_id	Unique identifier for pipeline execution
file_name	Original WSI name
new_file_name	Canonical renamed identifier
datamatrix	Raw barcode string (if available)
labid, year4, case6	Parsed CaseID components
pot, block, section	Specimen fields
stain	Normalized stain name
method_of_extraction	barcode or roi_ocr
inserted_at	UTC timestamp

9. Deployment & Reproducibility (Rewritten)

WSI-Babel-Shark supports reproducible execution through:

1. Native Python execution
   Each module exposes a CLI entrypoint (e.g., label run, dm run, extract run, pipeline run).
2. Watch-Folder Automation
   Continuously monitors the ingestion directory and triggers processing automatically.

3. Docker Deployment
   An official container (bioinfo2026/wsi-babel-shark:latest) encapsulates the full environment, including all Python dependencies, classifier prototypes, ROI sets, and default configurations.
   Canonical invocation:

```
docker run --rm \
  -v <WSI_folder>:/slides \
  -v <output_folder>:/output \
  bioinfo2026/wsi-babel-shark:latest \
pipeline run --config /app/config/config.docker.user.yaml
```

Docker guarantees version stability, dependency isolation, and platform-independent reproducibility across Linux, Windows, and macOS.

The orchestration layer ensures deterministic execution regardless of deployment method.

10. Version & Repository Information

- **Current version:** Babel-Shark v1.0 (2025)
- **Programming language:** Python 3.10+
- **Source repository (GitHub):** https://github.com/WSI-Babel-Shark
- **Docker Hub (official container):** https://hub.docker.com/r/bioinfo2026/wsi-babel-shark
- **License:** MIT
- **Contact:** aliyari.shahram@med.uni-heidelberg.de
